# An exploration of COVID-19 vaccination models for newcomer refugees and immigrants in Calgary, Canada

**DOI:** 10.1186/s13690-024-01255-y

**Published:** 2024-03-12

**Authors:** Fariba Aghajafari, Laurent Wall, Amanda M. Weightman, Alyssa Ness, Deidre Lake, Krishna Anupindi, Gayatri Moorthi, Bryan Kuk, Maria Santana, Annalee Coakley

**Affiliations:** 1 Department of Family Medicine, University of Calgary, Calgary, Canada; 2 Habitus Consulting Collective, Calgary, Canada; 3 Alberta International Medical Graduates Association, Calgary, Canada; 4 Department of Community Health Sciences, University of Calgary, Calgary, Canada

**Keywords:** COVID19, Vaccine, Models of vaccination, Refugees, Newcomers, Culturally responsive

## Abstract

**Background:**

The World Health Organization stresses the need for tailored COVID-19 models of vaccination to meet the needs of diverse populations and ultimately reach high rates of vaccination. However, little evidence exists on how COVID-19 models of vaccination operated in the novel context of the pandemic, how vulnerable populations, such as refugees, experience COVID-19 vaccination systems in high-income countries, and what lessons may be learned from vaccination efforts with vulnerable populations. To address this gap, this study explored COVID-19 vaccine delivery models available to newcomer refugees and immigrants, and refugee experiences across different COVID-19 vaccine delivery models in Calgary, Canada, and surrounding area in 2021 and 2022, to understand the barriers, strengths, and strategies of models to support access to COVID-19 vaccination for newcomer refugees and immigrants.

**Methods:**

Researchers conducted structured interviews with Government Assisted Refugees (*n* = 39), and semi-structured interviews with Privately Sponsored Refugees (*n* = 6), private refugee sponsors (*n* = 3), and stakeholders involved in vaccination systems (*n* = 13) in 2022. Thematic analysis was conducted to draw out themes related to barriers, strengths, and strategies of vaccine delivery models and the intersections with patient experiences.

**Results:**

Newcomer refugee and immigrant focused vaccination models and strategies were explored. They demonstrated how partnerships between organizations, multi-pronged approaches, and culturally responsive services were crucial to navigate ongoing and emergent factors, such as vaccine hesitancy, mandates, and other determinants of under-vaccination. Many vaccination models presented through interviews were not specific to refugees and included immigrants, temporary residents, ethnocultural community members, and other vulnerable populations in their design.

**Conclusions:**

Increasing COVID-19 vaccine uptake for newcomer refugees and immigrants, is complex and requires trust, ongoing information provision, and local partnerships to address ongoing and emerging factors. Three key policy implications were drawn. First, findings demonstrated the need for flexible funding to offer outreach, translation, cultural interpretation, and to meet the basic needs of patients prior to engaging in vaccinations. Second, the research showed that embedding culturally responsive strategies within services ensures community needs are met. Finally, collaborating with partners that reflect the diverse needs of communities is crucial for the success of any health efforts serving newcomers.

**Table:** 

Text box 1. Contributions to the literature
• Limited evidence exists on how COVID-19 models of vaccinations increase vaccine demand and uptake for vulnerable populations, such as refugees.
• COVID-19 models of vaccinations faced evolving factors over time, including changing public health information, mandates, eligibility criteria, funding limitations, and the need for advocacy. Specialized newcomer refugee and immigrant models relied on partnerships between diverse organizations to provide culturally responsive services and navigation supports.
• Vaccine interventions focused on newcomer refugees and immigrants should address basic needs of patients prior to vaccinations, include multiple touch points with patients through trusted personnel, and empower community partners to leverage their expertise, advocate, and shape services.

## Introduction

Early research on COVID-19 vaccine coverage in high-income countries found that immigrants and refugees were at increased risk of being under-vaccinated [1] and disproportionately impacted by the COVID-19 pandemic [2], yet their vaccine uptake was lower [3] and hesitancy was reported to be higher than in the general population [3]. Canada showed a similar trend with varied rates for population groups and legal statuses [2, 4–7]. Implications of newcomer refugee health disparities in Canada arose to mainstream awareness during the initial wave of COVID-19. An example was the meat packaging facility outbreak of 2020 near High River, Alberta, Canada, which was the largest outbreak in North America due to a single source, with over 1500 reported infections [8]. A culturally responsive model of care was leveraged through health and community partnerships in response to the outbreak. Learnings and partnerships from this experience became the foundation for Calgary area models of COVID-19 vaccination to newcomer immigrants, including refugees.

Models of vaccine delivery exist in contexts of vaccine hesitancy, vaccine confidence, and other factors such as trust in healthcare and the vaccine approval process [9]. Delays or refusal of vaccination are not solely the outcome of personal choice but other systemic factors such as structural racism and inequitable access [10]. Table 1 summarizes intersecting determinants of under-vaccination identified for migrant communities.

**Table 1:** Determinants of under-vaccination among migrant populations

Information inequities
• Limited access to health information leading to inadequate knowledge about vaccinations and vaccine-preventable diseases [11, 12]
• Language barriers and/or low literacy [10–13]
• Vaccine misinformation [3, 10, 14]
• Low health literacy [10, 15]
• Information not accessible or not provided in an acceptable format [11]
• Inadequacy of public health vaccination campaigns targeting migrant groups [16]
• Confusion related to vaccine eligibility [17]
Personal beliefs
• Low risk perception of vaccine-preventable disease [13]
• Vaccine-specific hesitancy (e.g. fears of adverse effects, vaccine safety concerns) [1, 11, 18]
• Ethnic, cultural, or religious values [1, 11, 12, 18]
Previous systems experiences
• Experiences of racism, discrimination, or social exclusion within health systems [1, 10, 11, 15, 19]
• Distrust of the health/medical system and/or government systems [10, 12, 15]
• Fear of accessing the healthcare system based on legal status (e.g., undocumented migrants) [14, 17]
Structural inadequacies
• Inadequate vaccine delivery coordination by public health authorities [11, 14]
• Gaps in national policies on migrant/refugee health services and vaccinations [1, 11, 19]
• Lack of vaccine coverage in the country of origin [14]
• Barriers or inequities to accessing healthcare in general that translate to vaccine access [1, 19]
Accessibility of vaccine services
• Lack of awareness and/or physical access to immunization services [11]
• Difficulties navigating the healthcare system [1, 19]
• Low digital literacy and lack of access to technology for booking and accessing information [11]
• Financial barriers accessing vaccination; including indirect costs such as taking time off work [1, 9, 11, 19]
Intersecting risk factors for under-vaccination
• Level of education [13]
• Income [1, 19]
• Family size, birth order of child [11]
• Age [7]
• Country/continent of origin [7, 20]
• Geographic location (e.g., rural/urban, neighbourhood) [7, 21]
• Faith and/or culture [1, 11, 12, 18]
• Legal status [14]

Since 2003, various vaccination models for newcomer refugee and immigrant populations have been reported in the health literature, primarily from the United States (*n* = 17) (Aghajafari, Guzek, Kamal, et al.: A scoping review of COVID-19 vaccination models for refugees, unpublished) with only two studies conducted in Canada [22, 23]. Vaccination models range in intervention scope and components – including wide-scale information campaigns [24, 25], mobile outreach programs [21, 22, 26, 27], and community vaccination sites [28, 29]. Most vaccination models incorporate multiple elements including vaccine promotion, coordination, and vaccine delivery, and some incorporate language translation (Aghajafari, et al.: A scoping review of COVID-19 vaccination models for refugees, unpublished). More recent vaccination models discussed in the literature involved some form of text messaging, email, or social media communication (Aghajafari, et al.: A scoping review of COVID-19 vaccination models for refugees, unpublished).

Research emphasizes the importance of health systems that prioritize high risk and excluded groups, including migrants, for vaccine delivery [1, 30]. Health agencies emphasize that standards and approaches be adapted to diverse contexts [11], attentive to diversity within migrant populations [30], and tailored to community specific barriers, beliefs, practices, and motivations [11, 30–33]. To increase COVID-19 vaccine coverage for newcomers specifically, systems must include accessible, low barrier, linguistically appropriate, and culturally responsive services [11, 17, 32, 34], tailored information campaigns [11, 13, 14, 17, 26, 30–33], and community consultations and non-governmental partnerships [10, 11, 14, 17, 31, 34].

Nuanced strategies to increase COVID-19 vaccination rates among newcomer refugee and immigrant communities in Western Canada are not well understood, with only two studies identified that were based out of Canada [22, 23]. Furthermore, what is generally known about refugee access may not be consistent in the novel COVID-19 context and strategies are not well reported. An opportunity to address gaps in local COVID-19 vaccination models for refugees and other vulnerable newcomers was initiated in Calgary in 2021, as soon as vaccines were available. This initiative included the Calgary Catholic Immigration Society (CCIS), the Alberta International Medical Graduates Association (AIMGA), and the Mosaic Refugee Health Clinic in Calgary. These organizations, who are research partners in this study, were at the forefront of several local vaccination efforts targeting newcomer refugees and immigrants. This study used qualitative methods to explore the strategies employed to achieve equitable COVID-19 vaccine coverage among newcomer refugees and other vulnerable immigrants in Calgary and area, in Spring 2021 through Fall 2022.

## Methods

### Research design

Researchers took an interpretivist approach to make sense of phenomena that were rapidly occurring and evolving during the COVID-19 public health emergency in 2021–2022. The goal was to explore multiple subjective perspectives “to uncover patterns of human activity, action, and meaning” [35], by drawing on the accounts of system patients, healthcare personnel, community partners, and vaccine advocates. In this study, due to multiple ways of interpreting and using terms such as newcomers, refugees, immigrants, and any compounds such as ‘newcomer refugees,’ we use the terms ‘newcomer refugees’, ‘refugees’, and ‘newcomer immigrants’ to refer to recently arrived refugees (less than three years in Canada), refugees in general, and recently arrived immigrants, respectively. For example, ‘newcomer refugees and immigrants’ refers to recently arrived refugees and immigrants with similar under-vaccination factors as refugees, such as those related to access and language. The purpose was to keep these labels in line with what participants shared.

The research team was composed of university faculty and consultants with previous experience conducting research on vaccinations for newcomers, partners involved in local vaccination models for newcomer refugees and immigrants, and first language data collectors. All persons had different strengths and levels of involvement. For example, one member was key at making connections, while others were designated for data collection. Data collectors came from diverse national backgrounds such as Canada, France, Ethiopia, Iran, Lebanon, United Arab Emirates, and Pakistan. Most had post-graduate degrees, and all shared an understanding of data collection, ethics, research procedures, and best practices conducting research in intercultural settings through group training. The core research team met monthly to facilitate group insights and momentum, and the inclusion of research partners from the community (CCIS, AIMGA, Mosaic Refugee Health Clinic) was critical to provide the team with real-time insights into the area’s evolving trends and add subtleties to the interpretation of findings. The research team was also deeply aware that most of the research team were not from the same social positions and cultural backgrounds as refugee participants, that participating in a research project was not the immediate concern for most refugee participants, and that the topic could be sensitive as it was immersed global sentiments around vaccination and COVID-19. To navigate these conditions the research team conducted regular debriefs to discuss aspects of the research, such as the effectiveness of recruitment, best practices with specific cultures, emerging concerns, and whether interview guides were helping interviewers learn more about the research questions. These regular check-ins and discussions not only facilitated communication, they also ensured that the research group had a consistent approach from recruitment to analysis.

### Sampling and recruitment

This exploratory research involved interviews with: Government Assisted Refugees (GARs, *n* = 39), Privately Sponsored Refugees (PSRs, *n* = 6), sponsors of (Privately Sponsored) refugees (*n* = 3), and key informants (stakeholders) involved in the design or delivery of COVID-19 vaccinations for newcomer refugees and immigrants (*n* = 13). Convenience, purposive, and snowball sampling were used to recruit study participants. Afghan GARs were recruited by convenience sampling facilitated by CCIS and AIMGA, immediately following their vaccination on-site at a temporary housing facility. Prior to vaccination, participants were offered a virtual information session in Dari and Pashto, introduced to the study purpose, given an opportunity to ask questions, and self-selected for participation. PSRs and Sponsors were recruited through purposive sampling by a CCIS staff. These clients were given information about the study and self-selected for participation. Key informants were identified by project partners, who supported making connections as needed. Additional key informants and persons from newcomer groups flagged by partners as vulnerable were solicited through snowball and purposive sampling but declined to participate. Consent for GAR interviews was verbal to build rapport through a paperless and less formal experience, and written for all others. Consent forms, scripts, and interview guides for refugees and sponsors were translated into first languages through certified translators and data collectors. All interview participant groups were provided a cash honorarium for their participation except for GAR participants, who had brief interviews, to reflect participants’ expertise and time commitment.

### Data collection

The project was approved by the University of Calgary’s Conjoint Health Research Ethics Board (CHREB), and researchers followed specific steps to respect and protect participants, and ensure confidentiality and anonymity where possible. For example, consent was broached patiently with refugees and room was given for any questions and concerns. Verbal consent was outlined in first-language and written consent forms were translated. Identifying information such as names were omitted in transcripts and all information was stored on secure servers. The research team was deeply aware of power imbalances between recently arrived GARs who had limited proficiency with English and researchers, the recency of their upheaval, and the impacts this may have on interviews, such as shortened responses. The team addressed this by working through trusted intermediaries, engaging first language data collectors, and hosting first-language information sessions about the study. The research team also took steps to meet GARS and PSRs where they were at, which included taking extra time prior to and after interviews to talk about matters not related to the study. These steps facilitated more comfortable conversations between refugees and interviewers, provided researchers with detailed answers, and helped researchers build credibility in the community.

Refugee and sponsor interviews were completed in first-language (Dari, Pashto, Arabic, and Amharic) with exceptions in English. Some interviews were conducted in a mix of English and first-language. GAR interviews (*n* = 39) were brief (5–10 min), structured, in-person interactions following vaccination at an on-site clinic for newly arrived GARs (less than three months in Canada) from Afghanistan. A minority of longer phone interviews (30–45 min) were also conducted in first-language with PSRs (*n* = 6) who had been in Canada for less than three years and more than 6 months, and with Sponsors (*n* = 3) of PSRs who aided with vaccinations. Key informant interviews were semi-structured (30–45 min) and completed by telephone or video conference, per participant preference. One interview included two participants, for a total of 13 key informants across 12 interviews. These participants included medical professionals (*n* = 2), public health representatives (*n* = 2), service provider organization (SPO) staff (*n* = 5), other staff (*n* = 1), community advocates (*n* = 2), and international medical graduates (IMGs) (*n* = 1).

The research team used a mix of structured and semi-structured interviews. Separate interview guides were created for all participant groups, including GARs, PSRs, Sponsors, and key informants. Interviews with refugees and sponsors primarily focused on past and current COVID-19 vaccination system experiences, concerns, barriers to access, and facilitating factors with varying levels of depth. The research team used a short, structured interview format for GARs to not burden them with too many questions, as they were very recent arrivals, and to support the comparison of answers between GAR participants. A longer, semi-structured interview guide was used for PSRs and Sponsors to explore in depth the experiences of refugees who had been in Canada longer. Interviews with key informants were also semi-structured and focused on descriptions of vaccination models, how they changed over time, strengths of models, barriers to vaccination, strategies to address barriers, trends with patients, and key learnings. As vaccination strategies were ongoing over the course of the study, interviews with key informants adapted to focus on emergent findings such as vaccine advocacy and specifics of models where details were sparse. Furthermore, later interviews with key informants became a time to reflect with key informants more broadly on the long-term mobilization of the COVID-19 vaccine in refugee and newcomer communities, and less on the impacts of working with refugees (e.g., staff gain increased understanding of cultural subtleties) or the practicalities of models of vaccinations, such as how a model set up intake and information provision, the barriers that models faced, and how models adapted. By the last interviews with refugees, sponsors and key informants, researchers felt that they had achieved a point of data saturation regarding newcomer refugee and immigrant models of vaccination in the Calgary area, whereby no novel findings on vaccination models were discussed.

Researchers initially sought out an equal number of men and women refugee participants to achieve as close to equal representation as possible. However, due to the practicalities of recruitment with intermediaries and self-selection for participation, the result was a greater proportion of men compared to women participants. Researchers initially started collecting key demographic markers from refugee and sponsor participants, such as gender, country of origin, and family status and composition. However, due to the difficulties of systematically collecting this information in a context of multiple data collectors, recruitment intermediaries, and multiple participant groups (e.g., an interviewer forgets to ask, the intermediary does not have that information, a participant does not answer the question), this information was not confirmed for all participants. Furthermore, the decision was made in consultation with partners to not burden recently arrived refugees with too many questions. This was done to be ethical with people who were forced to seek refuge in another country and facing new circumstances in Canada. It was also not practical to follow up with participants if certain information was not collected during the touch point.

### Data analysis

Interviews were audio-recorded or, if consent to record was not given, detailed notes were taken. All interviews were transcribed and if relevant translated to English. The team conducted inductive and deductive thematic analysis to draw out findings from interviews and ensure that themes were grounded in participant accounts and scholarship [35]. This allowed researchers to draw on multiple forms of interpretation to contextualize and push findings towards the central research focus. Specifically, these interpretations were informed by previous research and theories on health equity, vaccinations, and newcomer refugee and immigrant healthcare systems, researchers’ own experiences and expertise in the field of health and migration, and by the development of specific codes and themes based on knowledge and/or inductively identified themes in the data. The interplay of experience, induction, and deduction encouraged researchers to build thematic categories through multiple rounds of refinement, revisit relevant scholarship and data throughout to refine themes, and identify any further lines of inquiry. The final results were write-ups focused on how to frame specific social issues, themes or concepts, such as non-equitable COVID-19 vaccination systems for refugees in Calgary, and on addressing specific issues and themes, such as how to achieve equitable COVID-19 vaccine uptake for newcomer refugees and immigrants.

Researchers developed a code guide to sort through interview data and focus on barriers, strengths, and strategies of models from multiple perspectives. The guide was split between codes for patient groups, including GARs, PSRs and sponsors, and codes for key informants. Two sets of codes were used as patient groups discussed similar topics, despite having different interview tools. Refugee and sponsor codes were developed to capture previous migration experiences, concerns, and vaccination system experiences, including barriers, strengths, recommendations, reasons for refusing or delaying vaccination, and motivations for vaccination. The codes for key informants included codes for patient concerns, vaccination system descriptions, barriers to vaccinations for newcomers, strengths, learnings, and measures to facilitate vaccine access. To ensure inter-coder reliability and the robustness of codes, two members initially drafted a list of codes based on their knowledge and a close reading of separate transcripts from each participant group, and then converged their lists alongside a third member. The two researchers then piloted the entire guide on separate transcripts for all participant groups, discussed the pilot with other research team members, and made subsequent revisions to the guide. All interviews were coded by one member following guide validation. Throughout the process of code guide creation, coding, and theme building, researchers also engaged in discussion with the data collection team to understand their interpretations from a cross-cultural lens. This included discussing specific sections of text that could be interpreted in different ways, and the applicability of codes to sections of text and the inferences that could reasonably be drawn. Researchers used Microsoft Excel to sort through interview data and organize it by codes, as the software was ideal to facilitate collaboration between multiple group members. Specifically, it did not require significant training to use compared to most qualitative data management software, it was accessible to all members, and it allowed members to share ongoing progress with little effort. Microsoft Word was then used to review, organize, synthesize, and refine the emerging and final themes for the same reasons as Excel: members knew how to use it, it was accessible, and it allowed for easy sharing of materials.

## Findings

Table 2 below outlines participant characteristics. The research team recruited diverse key informants to learn about refugee specific vaccination systems in depth and breadth. At the time of data collections GARs accessible through CCIS were all from Afghanistan. Efforts were also made to speak to other groups of GARs however these efforts did not yield any participants. A small group of PSRs and Sponsors from diverse backgrounds were also recruited. Although efforts were made to systematically collect other demographic information such as age range and country of origin, the research team was not able to reliably compile this information for all participants. What was confirmed was that all 39 GARs originated from Afghanistan, some PSRs and Sponsors identified as being from Jordan or Ethiopia, and some did not specify. Key informants also reported that populations of concern were typically from racialized population groups, such as Arabs, West and East Africans, South Asians and Southeast Asians.

**Table 2:** Breakdown of participant characteristics by position, language, and gender, for key informants, refugees, and sponsors in Calgary, 2021–2022 (*N* = 61)

Key informant breakdown by position and gender (*n* = 13)								
Position in vaccination systems							Gender (n)	
Medical Professionals (n)	Public Health Representatives (n)		Immigrant Serving Agency Staff (n)	Community Advocates (n)	International Medical Graduates and Other Staff (n)			
							Men	Women
2	2		5	2	2		7	6
Refugee and sponsor breakdown by language and gender (*n* = 48)								
Participant type (n)	Language of interview (n)						Gender (n)	
	Dari	Pashto		Arabic	Amharic	English	Men	Women
GAR (39)	20	19					26	13
PSR (6)				6			4	2
Sponsor (3)				1	1	1	1	2

The following presents an overview of the explored COVID-19 vaccination models available to refugees (and in some cases other specific newcomer groups) in 2021–2022, followed by specific approaches used to target under-vaccination factors, such as barriers to access, lack of knowledge, and lack of information in first languages. This section focuses on solutions and approaches used by vaccination models in response to known gaps or barriers. For a more robust discussion on gaps in services and under-vaccination factors for newcomer groups, including refugees, see references in Table 1 and previous scholarship [1, 17, 18]. Importantly, while GARs, PSRs, and Sponsors spoke about vaccination models available to refugees, not all key informants were involved in vaccination models solely specific to refugees. Some models were specific only to unique population groups such as refugees or Temporary Foreign Workers (TFWs), while others were specific and available to broader population groups, such as newcomers in general, which included refugees. Additionally, while most models of vaccination were solely focused on delivering COVID-19 vaccines, some also provided a broader scope of public health services, such as pharmacies, physicians’ offices, and the Mosaic Refugee Health Clinic. Due to these subtleties from the data, our focus on refugees, and the consideration to protect the confidentiality of key informants in vaccination models, case examples are modestly fictionalized to protect anonymity, themes and solutions are presented as aggregates to demonstrate different types of COVID-19 vaccine delivery, and efforts are made to draw out findings for newcomer refugees and those with similar under-vaccination factors.

### Overview of COVID-19 models of vaccination

Vaccination options for newcomer refugees and immigrants included specialized models and mainstream models. All models were Calgary-based, except for two rural models in Southern Alberta. Specialized clinics were typically short-term, with only two examples of longer large-scale models. Mainstream vaccination options available to these groups included large clinics, pharmacies and physician clinics. Refugees could access vaccine services via Mosaic Refugee Health Clinic, a specialized model and clinic that provides comprehensive health care for recently arrived refugees. Below are anonymized examples of specialized models to provide context to the theme of addressing hesitancy and under-vaccination factors for newcomer refugees and those with similar under-vaccination factors, and its subthemes that follow.

Case example 1, mobile clinic: An immigrant-serving agency (ISA) program serving Temporary Foreign Workers (TFWs) identified that TFWs living in rural and remote locations were under-vaccinated due to language and transportation barriers, and lack of knowledge and healthcare numbers (a provincial identifier for healthcare coverage in Canada). Similarly, employers faced challenges in coordinating appointments to mainstream clinics and in facilitating transportation and time off for appointments. Due to these structural challenges and inconveniences, this group had a low likelihood of vaccination despite interest and conditions that put them at higher risk of COVID-19 outbreaks. A mobile vaccination clinic with a private clinic was established to provide 1st and 2nd doses to TFWs in rural locations during Summer 2021. Clinic sites (usually a farm) were coordinated with employers and neighbouring farms. On-site ISA staff provided interpretation and service connections. This clinic would have continued, however, funding was halted.

Case example 2, vaccination upon arrival: An ISA-run hotel used as temporary housing for Afghan GARs upon arrival hosted regular, on-site vaccine clinics. Clinics provided services to refugees as part of initial processing activities. The availability of on-site vaccination was critical during periods of mandated quarantine following international travel. Two iterations operated from early to late 2022. The first was led by a private clinic and the second by provincial services. Clinics engaged IMGs to host first-language information sessions, on-site information booths, and meet the ongoing needs of refugees. An on-site IMG shared how religious and cultural practices intersected with their approaches:

“I remember it was […] not only vaccination. We had Ramadan going on, so we had the Zoom meetings, and there were lots of [refugees] who attended those meetings on the importance of fasting and if they had diabetes, or they were pregnant or just so. I mean, there are many different health issues that are important, and they need to be addressed whenever, like a group of people are arriving.” (Key informant 08).

Case example 3, tailored community clinic: a partnership between a private medical company and non-governmental organizations operated from spring 2021 to spring 2022. This clinic provided low-barrier access for groups facing systemic health service barriers, including urban Indigenous, migrant, and unhoused individuals. Partnerships were tailored for access and cultural safety. A healthcare number was not a prerequisite to booking. Instead, numbers were produced on-site for those who did not have one.

Case example 4, large temporary community clinic: a partnership between the provincial health provider, grassroots organizations, and an ISA provided first-dose COVID-19 vaccinations to all eligible persons, with a focus on immigrant and ethnocultural communities. Grassroots organizations and ISAs conducted outreach and provided support to patients prior to the clinic and on-site. The clinic was strategically located to minimize geographic barriers. It operated with extended hours to accommodate diverse schedules and demands. The clinic was closed after one weekend.

### Addressing hesitancy and under-vaccination among newcomer refugees and immigrants in Calgary

#### COVID-19 and the evolving conditions of vaccinations

Table 3 identifies participant quotes related to subthemes of approaches to address hesitancy and under-vaccination factors. A central point from all participants was that vaccinations took place in evolving conditions. These conditions included COVID-19 case counts, vaccine availability, changes in public health policy (accepted vaccines, eligibility, mandates, and incentives), funding opportunities, media trends, and the overall political environment (see Table 4 for contextual factors along with verbatim quotes). Vaccine and quarantine mandates in particular shaped vaccination models and the experiences of patients. Two commonly cited reason for vaccination were health and travel. As one GAR shared, they received the vaccine “[n]ot to be infected with Covid virus again and also to be able to travel.” (GAR 24) At different periods, federal, provincial, municipal government, and employer/business mandates existed, requiring vaccination for employment, travel, and access to certain non-essential businesses and services. Most mandates ended by mid-2022. In this landscape, models required frequent shifts in administrative practices, outreach strategies, information distribution, design, and partnership decisions throughout roll-out or during any one vaccine model.

**Table 3:** Subthemes of approaches to address hesitancy and under-vaccination factors for newcomer refugees and immigrants in Calgary and area (2021-2022) with verbatim quotes

Subtheme	Quote
Vaccinations in evolving conditions	"We updated paperwork almost weekly…guidelines, paperwork…we had weekly meetings [to discuss] what was new, this is what's changed… ‘Okay, now ages 12 to 17 is approved for this’. ‘Now this one only counts’ …’now Moderna is only half dose for this age group’. ‘Oh, now we don't give Moderna under 30, unless they really want it because of myocarditis’. Like the information avalanche was unreal, like nothing I've ever worked in. But it was always updated, always communicated. Trying to make sure we were all using the most up-to-date information.” (Key informant 03)
Information mobilization and cultural interpretation	“By then, we were approaching the religious leaders, we're sending the message […] that this is not something bad it has nothing to do with their religion. […] [W]e were trying to do that with the help of the […] traditional religious leaders, so we were and most of the time we were conducting these vaccination processes at home and at the mosque, which is a holy place in the people, faster. So we will go into the mosques, […] using their loudspeaker using the sound of the Mullah or the religious person and talking with the people that there is a vaccine.” (Key informant 08)
Targeted community outreach	“Our system […], the health system in Alberta is not set up well to serve this population. So…having [an immigrant service agency partner] there, helping [refugees and newcomers] navigate the system and helping coordinate clinics and, you know, where to go and how to access it, and how to get a health number. You know, without an organization like that or a partnership like that, I would imagine it would be extremely difficult to be able to navigate the system.” (Key Informant 03)
Low barrier, community-based, culturally responsive clinic design	“We had extremely good partnerships [for specific population groups]. They called, they advertised… they found volunteers everywhere, went to houses, went to community, the churches, the yard sales. All those kinds of things and had signs and advertising. And so we had a ton of partners that did that work […] like we just had so many partners that did the work for us and got them in the door for us.” (Key informant 02) Referring to community partners: “I don't know how they organized it, but they would arrange groups or families and they would come with them, they would from start to finish. They would come to the door with them. We would get them registered. Lots of them, we had to create Unique Lifetime Identifier’s (ULI) for them if they hadn't had one created yet… their team would help support in terms of translation, all that stuff. And then we would help, we would funnel them through the clinic right and get them registered and vaccinated and aftercare.” (Key informant 02)
Partnerships with NGOs	“We had extremely good partnerships [for specific population groups]. They called, they advertised… they found volunteers everywhere, went to houses, went to community, the churches, the yard sales. All those kinds of things and had signs and advertising. And so we had a ton of partners that did that work […] like we just had so many partners that did the work for us and got them in the door for us.” (Key informant 02) Referring to community partners: “I don't know how they organized it, but they would arrange groups or families and they would come with them, they would from start to finish. They would come to the door with them. We would get them registered. Lots of them, we had to create Unique Lifetime Identifier’s (ULI) for them if they hadn't had one created yet… their team would help support in terms of translation, all that stuff. And then we would help, we would funnel them through the clinic right and get them registered and vaccinated and aftercare.” (Key informant 02)
Flexible funding	“There's a shared complexity in even accessing emergency funding that we knew could support people. There are the bureaucrats that are involved, and then, of course, the challenges of the inaccessibility of this funding, just because of how things have been structured, and particularly, for newcomers and refugees because that is always an issue. If you see how resources flow, you realize that they don't necessarily flow very well to newcomer and issues.” (Key informant 10)
Other factors to address	“If we are not compassionate enough, and we are unable to understand them… vaccination probably is only taking a few minutes to be done, but in long term we are going to lose that trust. These groups of people are very traumatized. Their mental well-being is not really the best, and if we are unable as a health care provider, we are unable to detect that, and address that.. they wouldn't be very enthusiastic to get the vaccine. Not only the [COVID-19] vaccine, it could be for anything else in the future.” (Key informant 08) “It is helping people where they're at but also helping them understand what we can do to help them get better health outcomes. So, [refugees are] a really marginalized population generally, and this is where public health really adds a lot of value, I think, and helps people navigate the healthcare system and engages them in healthcare and develops trust with this population because they've been through a lot often. So establishing trust with health authorities sometimes takes a long time, but they're very appreciative and it helps them trust in our system basically.” (Key informant 12) "How fun or engaging or comfortable something is matters too right. At the end of it.. you can watch like [SPO] staff just talking to people all the time, all the stuff …that's high intensity work right?” (Key informant 13) “[…] to get them engaged and get them to understand how the system works in Calgary and then working towards them coming to our routine clinics, so that we can support them where they live in the community. Initially they're kind of centralized in one area, but then they spread throughout the zone and other parts of the province, and so we want to kind of start that model of getting them to connect with supports in the community and support accessing health services within the way it's normally done within Alberta.” (Key informant 12) “They already went through [so] much. They went through a lot, so we just want to make their life easier here [by explaining what COVID-19 vaccination or no COVID-19 vaccination means]. Sometimes very small, tiny things make a huge difference, yeah?” (Key informant 01) “Alberta was relatively limited in its deployment of community-based vaccination clinics compared to other provinces right. You know the vaccine role in Alberta is primarily driven initially by pharmacies and public health. Later on primary care like family doctors are giving them permission to get vaccines and then later, later on, like we are able to do these mobile vaccination clinics through a medical [provider] all over the province, but we are much lower than the other provinces in terms of just like I guess like vaccine deployment.” (Key informant 13) "We can be available after hours… that's what we should target if people are working in bigger factories or institutions, hospitals or other places. We can go there and do vaccination…With the kids' vaccination, it was such a low rate because nobody wants to do anything differently… No approval came through for schools [even though these] are good places for vaccination.” (Key informant 11) “There are lots of wastage of vaccines which can be prevented, if we have a central kind of approach. Or we have different facilities, who are vaccinating that can talk to each other, or they can have a centralized approach…. why [are we] wasting when the rest of the world doesn't have it and there's so much shortage everywhere.” (Key informant 11)

**Table 4:** Contextual factors impacting COVID-19 vaccine coverage for newcomer refugees and immigrants in Calgary and area (2021–2022) with verbatim quotes

Description of contextual factors	Verbatim quote
Vaccine supply: Availability of vaccines was a common challenge early in the vaccine roll-out especially, meaning some clients would arrive to appointments only to find out there were no vaccines available. At other times, vaccines were in oversupply and discarded.	“There were periods when vaccines were in short supply or certain vaccines were in short supply, which meant people were sometimes not able to receive their vaccine even when they booked it or had to wait to receive their preferred dose. Similarly, when vaccines were more widely available, some of the need for the more dedicated community models waned.” (Key informant 01)
Mandates: During most of the vaccine roll-out period explored, vaccine mandates were in place requiring vaccination for air travel, border crossings, some employment, and some access to facilities. Informants found that this encouraged vaccine uptake but did not necessarily mitigate concerns around vaccine safety.	"Vaccine mandates.. stimulated demand again and so we saw a big spike in demand when the [Restrictions Exemption Program] came in.. after that initial peak it plateaued again.” (Key informant 13)
Accepted vaccines: What vaccines were acceptable at any given time varied throughout the vaccine roll-out period as new vaccines were being developed and new research being released. This had implications for newcomers who sometimes were vaccinated in a previous country but that vaccine may not have been accepted. Therefore, an individual may have been defined as un-vaccinated in Canada but they had been vaccinated with a non-accepted vaccine. The changing rule around accepted vaccines was also a source of confusion for community members.	“[…] so many people took Sinopharm, so many people took Johnson and Johnson [prior to being with our clinic]. And the system just said no, this is not something legit or not something approved by our health care system. You have to take a full new series, either Moderna or you have to take Pfizer. In just like 2–3 weeks after that, they put on their website like this is all OK.” (Key informant 01)
Eligibility: Who was available for what vaccine doses changed frequently throughout the vaccine rollout. Therefore, vaccine uptake should be considered in the context of what was available and for whom at any given time. When new groups became eligible or new doses became available, demand would typically increase. Informants noted that when a new demographic group became eligible, for example, youth, there was also an immediate uptick in vaccination rates.	“When Omicron came… we expanded third doses to everybody, there was again an absolute surging demand over supply…” (Key informant 13)
Novelty of vaccine availability: Informants also observed a pattern related to information needs, hesitancies, and demand that related to the pattern of the vaccine rollout itself, and in some ways to what might be typical for when any new vaccine and medicine comes to be available.	“Questions and concerns changed over time as public knowledge shifted. For example, ‘what is it’ ‘I’m not sure..’ was prevalent in the beginning. Then it shifted towards questions and concerns about documentation, old records, QR codes, Alberta health equivalents, recognition of old vaccines.” (Key informant 13) In reference to late 2021 and early 2022: “It's higher than the early groups, because again, now everything is on track. Everything is smoother. Most of the people that we are receiving now, they have Pfizer vaccine or J&J. Most of them, either the kids already took Pfizer or they are going to start here.” (Key Informant 01)
Time of arrival and pre-arrival country(ies) of residence: For incoming newcomers, time of arrival to Canada (in relation to phase of vaccine rollout) and country (or countries) of origin/last residence was also a critical contextual factor. For example, key informants noted that there were more Afghan newcomers needing vaccines in August 2022 (first wave of arrivals to Canada) than a few months later, because those later groups had been able to be vaccinated in the country they were temporarily residing prior to arriving to Canada.	“The type of clients that we're seeing, and where they are in the process has changed.. there's more people who are fully vaccinated [now in mid-2022].” (Key informant 08)
Phase of vaccine roll-out: Similar to trends related to the novelty of vaccine availability, informants observed an increase to information needs, hesitancies, and demand that related to the accelerating vaccine rollout itself, and in some ways to what might be typical for any time a new vaccine, medicine, or otherwise comes to market. Informants discussed that some of the ebbs and flows in demand and interest/disinterest were not necessarily novel to COVID-19. They also noted that processes became smoother as personnel gained experience with specific vaccination models and populations.	“Questions and concerns changed over time as public knowledge shifted. For example, ‘what is it’ ‘I’m not sure..’ was prevalent in the beginning. Then it shifted towards questions and concerns about documentation, old records, QR codes, Alberta health equivalents, recognition of old vaccines.” (Key informant 13) In reference to late 2021 and early 2022: “It's higher than the early groups, because again, now everything is on track. Everything is smoother. Most of the people that we are receiving now, they have Pfizer vaccine or J&J. Most of them, either the kids already took Pfizer or they are going to start here.” (Key informant 01) “We got better at streamlining the process. You know we with the refugees in particular You know a lot of them came from large families, so instead of having three or four people sitting in the chairs, you explain things—you might have 12—so you might have grandparents and cousins so we would still kind of look at them as a group, you know they can ask questions back and forth, and before they ever you know decided what they wanted to have as far as immunizations so that definitely changed, that was a unique approach with them. The translators got pretty skilled at the questions so that became more streamlined for sure. And we found that too, we did some temporary foreign worker clinics in the summer, some outreach clinics there and same thing with the translators after a few clinics. You know they knew what to expect so they were able to streamline the process for us.” (Key informant 03)

#### Information mobilization and cultural interpretation

Key informants recommended that ample and comprehensive information reach communities ongoingly to promote vaccinations and address hesitancy. GARs and PSRs, on the other hand, routinely shared that personnel such as case managers, IMGs, nurses, and doctors, were helpful when they gave patients time to answer questions and were open about vaccine side effects. A primary strategy for specialized vaccination models was to provide accurate information through trusted networks and in first languages. The relationship between the source and the community impacted the way information was received and acted upon. All participants groups shared that faith leaders, native-language speakers, and IMGs were ideal candidates for this. Such figures could provide resources and referrals to services and support in a timely manner to their respective communities. Community members were more likely to act upon recommendations due to the trusted status of mobilizers, and the ability of candidates to deliver information through culturally appropriate frameworks.

Non-governmental organizations (NGOs) were effective in disseminating first-language information as they were nimbler. Examples included: timely translation of public health information into key languages, distribution of materials in community sites and SPO programs, online vaccine information sessions, and information booths at vaccine clinics. First language information and services were common, and many models embedded first-language staff or interpreters. Key informants discussed the importance of cultural interpretation alongside first-language services. This meant the translation of information was conducted in culturally relevant ways, such as those sensitive to faith or gender norms. Key informants emphasized the value of being cognizant of patient pre-and post-migration experiences and diverse cultural perspectives on vaccination when engaging in outreach and information provision.

#### Targeted community outreach

All key informants highlighted that outreach positively influenced newcomer refugee and immigrant vaccinations. Outreach was common for community partners, who leveraged existing connections and relationships of trust to mobilize information and connect newcomers to vaccination appointments. Key informants discussed the importance of making direct connections between potential patients and vaccine clinics as a strategy to address hesitancy and/or to mitigate barriers to clinic access. Trust was built through multiple points. During a COVID-19 case spike one model, who partnered with Case Example 4 by conducting outreach and facilitating access to vaccinations for various newcomer groups, including refugees, advocated that basic needs packages be delivered to address the immediate needs of residents before approaching residents about vaccines. This resulted in culturally appropriate food hampers being delivered to communities, and a relationship was built with community leaders who were able to provide timely information for vaccination efforts. This strategy was also used for TFW-specific models and services.

#### Low barrier, community-based, culturally responsive clinic design

Tailored and adaptable service delivery was another feature of refugee and newcomer vaccination models. This included community-based locations to provide vaccinations where people might already be gathered (e.g., cultural centre) or accessible locations. Design features included evening and weekend hours, diverse booking options (online, phone, and walk-in), attention to clinic atmosphere and efficiency, private rooms, and family booking options. Models leveraged partnerships to have personnel on-site to support navigation, provide interpretation, and make peer connections. Table 5 provides a summary of the various strategies used to integrate low-barrier, culturally responsive design along with verbatim quotations.

**Table 5:** Descriptions of low-barrier, culturally responsive COVID-19 vaccination services for newcomer refugees and immigrants in Calgary and area (2021–2022) with verbatim quotes

Descriptions of types and characteristics of low-barrier, culturally responsive vaccination services	Verbatim quotes
Community locations and on-site vaccination services: Key informants emphasized that refugees and newcomers are frequently based with barriers to access in the form of geographic and transportation issues. As a result, populations who wanted to be vaccinated were unable to reach clinics because public transportation was unreliable, irregular and not well-connected. Informants and clients also lamented the political inflexibility to offer vaccines in accessible locations such as schools or nearby clinics. Geography was also a main barrier for rural, TFWs	“Well, the biggest thing was that we were located at a hotel where a lot of the refugees were, that was their first stop in Calgary and Canada. So, the access, the convenience was right there; we were at their fingertips. So most of the refugees came through the clinic right at the hotel and then you know the ones that were hosted at a different hotel we ended up going to them. [We used] one of the conference rooms, so that they wouldn't have to bus or coordinate rides or transit anything like that.” (Key informant 03) “We went to various spots like [example of a cultural centre] and did it in their setting with their support with translation and them helping, [to] find all the people that needed it and get them through.” (Key informant 02)
Evening and weekend hours: Informants emphasized that newcomers are likely to be working outside of traditional hours or be unable to afford time off for vaccine appointments, or have employment that does not allow it. Therefore, flexible hours and evening and weekend availability for appointments was a primary strategy to increase access	“People are working hard and hours are different. They start work at seven in the morning till seven in the evening, so they can't go to the clinics. And that's why it was decided that going to a place where the people are, or where the clients are, to give vaccination, that'll be the best thing… Similarly at [a large leisure centre] registration was done over the weekend, so they were open […] from nine-to-nine Saturday, Sunday…As far as [my] clinic is concerned, we have normal hours but I did in my clinic some Saturdays or Sundays solely for vaccination. We booked right away.” (Key informant 11) “I think their main barriers, as I mentioned earlier, are timings for people whether they can get in or not. [If someone] has a bigger family, two or three generations in one home and how to get them and if one person drives and he can bring other people, especially weekend work better for them or after hours, that was the best thing for them.” (Key informant 11)
Attention to clinic atmosphere: Recognizing the importance of a positive clinic experience, and the potential for past system trauma, models were designed with patient comfort in mind. Modified elements included imagery, seating, wayfinding and signage (from the parking lot to the interior), staff layout, music, entertainment for children, and other elements related to the ambiance	“We had music playing all the time, we had a video screen up, we created an environment that was very calming. Families could sit together at big tables. Sometimes we had ten chairs at one vaccinator’s table, and [the nurse] would vaccinate all ten family members together. We didn't want to be inefficient, but we wanted to be welcoming and so there was a very strategic design to make that happen and to make it feel comfortable. We spread out the appointments so […] you never had to wait very long. You got greeted in a parking lot and shown where to park and then you got walked to the door and then you got walked to the next point. There was always a human to guide you through.” (Key informant 02)
Culturally responsive staff: Model partners hired and/or assigned key personnel and volunteers based on their lived experience and language spoken, to specifically work with newcomer and refugee groups (T2, Q31). This supported trust-building and the linguistic and cultural translation of medical information	“I think what's been like we've been lucky that we've always had people [internally] who speak the language so as an agency we're very diverse. And so I think just the nature of working in such an environment you learn how to be a little bit more culturally sensitive and you also learn from other cultures, because your colleagues are from the same cultures that your clients are from.. So they really understood the clients, they understood what they had been through.” (Key informant 06)
First language information and service delivery: Models provided written and oral translation services to meet the needs of patients (T2, Q32), through means such as certified health translators by phone, on-site staff and volunteers, vaccine navigators and various technologies such as Google Translate. The preferred services for staff and refugees were trained face-to-face translators such as vaccine navigators. Models provided materials and services to patients before and after receiving vaccinations in multiple languages. These could be in face-to-face and/or virtual settings	“You have people, imagine you have a 60-year-old man that does not speak English, and somehow, he ended up in Canada and he cannot go to the clinic. It's difficult for him. And learning a new language is not achievable that much for him, he just can't do it. It's just age and whatever is going on, it's not an achievable thing. They are not understanding. […] Yeah, that's why at the beginning, I always keep them (interpreters) for the vaccination. I keep with me at least 2 interpreters, 2 to 3 interpreters must be there.” (Key informant 01)
Other examples of culturally responsive design: • Availability of private rooms for vaccine administration • Availability of same-gender doctor/nurse • Whole-family appointments • Same-gender nurses available • On-site community service booths • Integration of vaccine navigators	
Other examples of low barrier design: • Provision of transportation to clinic, leveraging the capacity of NGOs • Clinics in locations accessible by public transportation • Provision of health care numbers on site (rather than requiring a number for booking) • Mix of booking options including telephone booking, online booking, and walk-in appointment • Capacity to make bookings for groups/ families instead of one by one • Booking support • Appointment reminder calls • Integration of vaccine navigators	

#### Partnerships with NGOs

Key informants considered non-governmental partnerships imperative for successful strategies for newcomer communities. Examples included partnerships between health services and one or more ISAs, community associations, ethnocultural associations, faith organizations, IMGs, and other non-profit service providing organizations. In general, the health service partner provided clinical expertise and services while community partners leveraged their position to conduct targeted outreach, integrate cultural responsiveness and cultural safety into service design, anticipate barriers, facilitate access, identify, and facilitate community-based opportunities, mobilize volunteers, provide interpretation, and disseminate information.

#### Flexible funding

Funding, which predominantly originated from public sources, was identified by key informants as a key factor shaping vaccination models. Provincial funding limitations meant that only some models were able to adapt and mobilize to specific community needs, and most models leveraged several sources of funding. Key informants highlighted that significant efforts were needed to advocate for flexible funding and that unless funders strengthen their community focus, the effectiveness of vaccination models will remain limited. Key informants emphasized funds are needed for translation, communication, public engagement, extended or flexible clinic hours, subsidized transport, and flexible locations of vaccination sites, and to reduce barriers, increase digital access, and fund community bridge builders.

#### Other factors

Key informants framed the importance of attending to user experiences to build patient trust in health services. Creating a positive vaccine experience not only helped vaccination outcomes, it fostered future use of the health system. Key informants focused on providing accurate information, respecting a patients’ decision to be vaccinated or not, and framed their interactions with refugees as a dialogue, as opposed to a more coercive approach. As one ISA staff emphasized: “It’s not convincing, it's getting consent [to be vaccinated].” (Key informant 07) Key informants highlighted that outreach and information provision required a compassionate approach sensitive to context. Knowledge of different belief systems, the resettlement process, and intersecting factors were helpful when addressing vaccine hesitancy. These capacities were often held by ISA staff. One vaccine advocate shared: “Individuals of [grassroots and community] organizations, they have the capacity. Not just the linguistic capacities. They also have the cultural abilities. They had the knowledge of their communities. They knew exactly what needed to be done.” (Key informant 10) Key informants emphasized the need to contextualize perspectives and hesitancies in the context of other demands, needs, resettlement journeys, and experiences with health systems, and adapt services to such contexts.

Outreach with community partners complemented the work of vaccine providers, and helped clinical providers better understand community members and adapt accordingly. One public health administrator highlighted multiple benefits of working with outreach partners:

“Doing outreach we learned a lot about outreach…and how to make sure we are where the population needs us to [go to them] rather than asking them to come to us. Especially with the refugee population, it's very difficult for them to navigate the current healthcare system: coming to a site that they have no idea where [anything] is, in a large city, and they have no idea of how to get around. It puts them at risk, and so, working with them, I think, is really where we need to go with this.” (Key informant 12).

A novel finding from this study was that vaccination hesitancies can persist after accepting a vaccine. For example, a key informant engaged in outreach calls with newcomer refugees early in the rollout described that many refugees who had already been vaccinated had unanswered questions and unaddressed concerns.

Models and adaptations were influenced by political context and government allowances. One challenge was the lack of timely changes in vaccination procedures to meet demand and adapt to changing contexts. The health system struggled to address structural barriers to access, which meant that communities struggled to catch-up with population-level vaccination rates. Key informants discussed limitations placed on approved locations (e.g., unable to vaccinate children in schools), restrictions on allowances/timings to provide booster doses, over and under supply of vaccines, limited government funding for partnerships or tailored services, and overall inflexibility to diversify options. The lack of system flexibility and readiness meant that informants could not preemptively address known barriers to access without advocacy and negotiations with officials to change vaccination practices.

## Discussion

This research found that vaccine hesitancy is complex [11] and simultaneously addressing individual, community, and structural factors of vaccination for newcomer refugees and immigrants is paramount [1, 19]. This paper discussed key features of newcomer refugee and immigrant COVID-19 vaccination models in Calgary and area during the 2021–2022 vaccine roll out. Strategies to facilitate vaccinations included: information mobilization, cultural and language interpretation, low barrier services, culturally responsive designs, outreach, non-governmental partnerships, and flexible funding. Other major factors in local newcomer refugee and immigrant vaccination models included sensitivity to patient experiences, flexible processes to surmount systemic challenges, and reliance on partnerships and community-led models. Partnership with community organizations enhanced staff capacity for effective communication, built trust at multiple touch points, and had targeted community engagement strategies. Where mainstream public health services lacked a tailored approach for unique populations such as refugees, community-based organizations and partnerships used their expertise and living experience to adapt to population specific contexts and health outcomes. A critical finding was the emergent role of NGOs in mobilizing information, affecting outreach, offering crucial support services, facilitating access, and embedding principles of cultural responsiveness into service design. Diverse organizations addressed gaps in vaccine information and delivery. Furthermore, IMGs provided medically accurate information in culturally responsive ways. While not all local vaccination models engaged in advocacy, vaccinations took place at no cost to patients and regardless of immigrant status in the Calgary area. Newcomer refugee and immigrant COVID-19 vaccination models were built as separate or ad-hoc streams for these specific populations. In a novel and quickly moving vaccine roll-out, these specialized vaccination models had a significant impact – community members who were vaccinated may have not accessed a vaccine or gained vaccine confidence otherwise.

Local vaccination models for newcomer refugees and immigrants in Calgary illustrated parallels to literature and the WHO’s actions to strengthen COVID-19 vaccine demand and uptake (see Table 6 in Appendix) [1, 11, 30–33]. Several contextualized models in the health literature have also enhanced vaccine uptake among similar underserved populations. Such vaccination models employed multipronged strategies similar to those used in Calgary, including intersectoral collaboration, translated communication through the right channels, building trust, and performing outreach [26, 27, 29, 36]. Only one model in the literature considered the integration of community partner input in policy, planning and decision making, as was found in Calgary area newcomer refugee and immigrant vaccination models [37]. Importantly, integrating community partner input was regarded as a key vaccination strategy with benefits for public health systems and newcomer refugee and immigrant patients.

Our study contributes to scholarship regarding refugee patient experiences at various system touch points. Vaccine clinics, immigration experiences, and outreach efforts are an opportunity to create trust with the local health system [21, 27]. Refugees and vulnerable immigrants are unlikely to access the health system if trust is not embedded as a core philosophy in health care delivery. Trust was fostered through multiple, concurrent strategies in local refugee vaccination models. This had a compounding effect on the relationship between community and the local public health system.

Another key finding was the impact that vaccination models had on health sector relationships, which were highlighted as potential barriers for newcomer focused vaccination models. While NGO partnerships are not new for the public health system, the extent of the involvement and impact of these partners was unique. Vaccination models relied on community partners to make inroads into hard-to-reach and hesitant communities. Partners were no longer limited to downstream actions – they played a crucial role in shaping upstream decisions around vaccine policy, funding, and accessibility. This empowerment of community partners to leverage their expertise and shape policy decisions and services is an aim within the field of global public health [38]. The essential public health function of health promotion and influencing health policy also aspires to empower communities [39, 40]. While this was cited as a strength of Calgary newcomer refugee and immigrant vaccination models, a key concern of informants was that contrary to the recommendation to permanently adopt COVID-19 policies and practices for equitable health outcomes [11], most vaccine funding streams were temporary.

Lastly, discussions around refugee vaccinations often overlook the basic needs of patients – needs that play a key role in health decisions. Our study underscored the importance of addressing the basic needs of newcomer refugee communities as a first step in health service delivery, especially for those who live in precarious conditions. Until the health system builds strong connections to community organizations and embeds them into policies and structures that address social determinants of health, achieving vaccine equity will remain a challenge. Additionally, participants shared examples of patient vaccine hesitancy even after accepting a vaccine. This was in part the result of rapidly evolving vaccine approvals and safety information in the scientific and public sphere, and prevailing mandates for work, travel, and access to parts of civil society. These factors led some people to be vaccinated despite feeling hesitant. Findings were suggestive of the need for concurrent or post-vaccination information services to address hesitancy that can persist or emerge after vaccination, especially in the context of a novel vaccine.

This study has specific limitations. It was exploratory in nature and is not an exhaustive view of vaccine responses in the Calgary area or a thorough case study of any one model. Nor were models and strategies evaluated for their effectiveness. Successes and recommendations presented are based on the experiences of authorized immigrants and refugees and stakeholders in vaccination systems. The partnership with CCIS and AIMGA shaped a bias towards information, data, and research participants connected to these organizations and vaccination models. The engagement of partners, while a potential source of bias, was ultimately viewed as a strength as it facilitated access to information, data, and populations that would otherwise have been difficult to tap into.

## Conclusion

Increasing COVID-19 vaccine uptake for newcomer refugees and immigrants is complex and requires trust, ongoing information provision, and local partnerships to address ongoing and emerging factors. Recommendations for system strengthening to increase vaccine demand and uptake of the COVID-19 vaccine for newcomer refugees and immigrants include ensuring flexible funding to meet population specific needs, such as open funding streams for pre-established partnerships between newcomer or refugee organizations and health care providing partners. Next, embedding culturally responsive practices and approaches within health care delivery ensures community needs are met. These efforts need to be sustained to make long-term impacts. Examples include clinic design, translated information, drawing on grassroots organizations, and embedding trust across service touchpoints. Finally, collaborating with partners that reflect the diverse needs of communities, including those that address basic needs, is crucial for the success of any community-based health efforts serving newcomers. These relationships must be based on equitable relationships that allow for community partners to co-develop services or approaches. By creating a collective model with a decision-making process driven by evidence and community input, the system can begin to bridge the divide and create trust within communities.

## Data Availability

The datasets analysed during the current study are not publicly available but are available from the corresponding author on reasonable request.

## Appendix

**Table:** Alignment of models of vaccinations to WHO priorities to strengthen COVID-19 vaccine demand and uptake for refugees and migrants

**WHO priority actions to strengthen demand and uptake** [25]	**Examples from models of vaccination**
Be driven by data	• Previous culturally responsive COVID-19 vaccination responses provided the foundation for new local models of vaccination • ISAs and healthcare service providers work with various levels of government to monitor population estimates and resource allocation
Coordinate, plan and implement	• Community partners are engaged in building and delivering various components of vaccination services, such as model design, communication strategies, adaptations and contingencies • Community engagement, outreach, and information provision is led by community organizations and ISAs • Models of vaccination adapt in real-time to changing needs of populations, such as information needs and outreach challenges
Address key barriers to health and vaccination systems	• Models provide low barrier, culturally responsive vaccine services, through on-site, mobile and strategically located services • Vaccinations are provided at no-cost and consent is broached in respectful manners • Personnel of various backgrounds are included, such as women, first-language speakers, and representatives of various ethnocultural groups
Ensure effective communication and build trust	• Models provide ongoing culturally and linguistically appropriate information through community partners and ISAs • Communication with community members is conducted by trusted personnel such as IMGs and ISA staff, on an ongoing basis • Positive patient experiences are tailored at various patient touch points • Feedback mechanisms give real-time information related to the needs of community patients
Monitor and respond to social media	• Social media is included in communication strategies, and strategies also include face-to-face and phone communication • IMGs are trained and respond in real-time to health and vaccine concerns through all platforms • Community feedback is collected through internal and external channels
Ensure effective community engagement	• Community-led responses incorporate trusted figures, such as grassroots leaders, religious figures, IMGs and volunteers • Community action plans are developed in consultation with ISAs, community organizations, and local leaders • Community members and volunteers are involved in disseminating information related to access and vaccines
Reinforce capacity and local solutions	• Healthcare workers build capacity to deliver newcomer and refugee services through collaborations with partners • Partnerships are tailored to provide services in various geographic zones • IMGs are trained as support personnel and vaccine navigators, and mobilized in various contexts • ISAs and community partners provide real-time feedback for services, and services adapt to feedback
Monitor, learn and evaluate	• Trends related to population uptake, hesitancy, and coverage are monitored by staff • Internal evaluations monitor progress of vaccination models • Models respond to emerging needs and population trends
